# Plasma circulating tumour DNA is a better source for diagnosis and mutational analysis of IVLBCL than tissue DNA

**DOI:** 10.1111/jcmm.18576

**Published:** 2024-07-25

**Authors:** Chao Chen, Yiao Di, Zhe Zhuang, Hao Cai, Congwei Jia, Wei Wang, Danqing Zhao, Chong Wei, Wei Zhang, Daobin Zhou, Yan Zhang

**Affiliations:** ^1^ Department of Hematology, Peking Union Medical College Hospital Chinese Academy of Medical Sciences and Peking Union Medical College Beijing China; ^2^ Department of Pathology, Peking Union Medical College Hospital Chinese Academy of Medical Sciences and Peking Union Medical College Beijing China

**Keywords:** ctDNA, intravascular large B‐cell lymphoma, mutational profile

## Abstract

Diagnosis of intravascular large B‐cell lymphoma (IVLBCL) is a challenge due to its heterogeneous clinical presentation and lack of specific markers. This retrospective study investigated the utility of circulating tumour DNA (ctDNA) sequencing for diagnosing IVLBCL and analysing its mutation landscape. A cohort of 34 IVLBCL patients enrolled and underwent plasma ctDNA targeted sequencing. The median plasma ctDNA concentration was 135.0 ng/mL, significantly higher than that in diffuse large B‐cell lymphoma (DLBCL) controls. Correlations were found between ctDNA concentration and disease severity indicators, LDH and SF. Mutation analysis revealed frequent mutations in B‐cell receptor and NF‐κB signalling pathways, including *MYD88* (56%), *CD79B* (44%), *TNFAIP3* (38%) and *IRF4* (29%). CNS involvement was significantly related with BCL6 and CD58 mutation. Patients with complicated hemophagocytic lymphohistiocytosis had significantly higher mutation rates in *B2M*. Comparison with DLBCL subtypes showed distinctive mutation profiles in IVLBCL. Moreover, plasma ctDNA detected more mutations with higher variant allele fraction than tissue DNA, suggesting its superiority in sensitivity and accessibility. Dynamic monitoring of ctDNA during treatment correlated with therapeutic responses. This study revealed the role of ctDNA in IVLBCL diagnosis, mutation analysis, and treatment monitoring, offering a promising avenue for improving patient diagnosis in this rare lymphoma subtype.

## 1 INTRODUCTION

Intravascular large B‐cell lymphoma (IVLBCL) is a rare subtype of extranodal diffuse large B‐cell lymphoma (DLBCL), named for the loose distribution of lymphoma cells within small to medium‐sized blood vessels.^1^ Unlike solid tumours, IVLBCL usually does not form a solid mass.^2^, ^3^ Also due to the near‐ubiquitous presence of the lymphoma cells, the clinical symptoms are highly heterogeneous. IVLBCL cells have strong and constant CD20 expression. CD10 and BCL‐6 were expressed in 22‐26% of Japanese cases.^4^ A LYSA study reported that BCL2 was expressed in 87% of patients and 43% of patients had a MYC/BCL2 double expression.^5^ Moreover, CD5 expression exists in 22–52% cases of IVLBCL,^6^, ^7^, ^8^ which is detected in only 5% of DLBCL cases.^9^ In conclusion, immunophenotype of IVLBCL displays the type of non‐GCB subtype. In terms of cytogenetics, the targeted next‐generation result showed that 44% and 26% of IVLBCL cases had *MYD88 L265P* and *CD79b Y196* mutations.^10^

The lack of specific characteristic symptoms, physical signs and the deficiency of specific tumour markers make IVLBCL difficult to be diagnosed. Furthermore, the rapid progression of IVLBCL significantly impact treatment outcomes if diagnosis is delayed.

The diagnosis of IVLBCL necessitates confirmation through histopathological biopsy, although the sparse presence of IVLBCL cells in small vessels in biopsy specimens presents diagnostic challenges.^11^ As a result, definitive diagnosis often requires multiple and repetitive biopsies. Nowadays, liquid biopsy using circulating tumour DNA (ctDNA) sequencing is increasingly utilized across various types of tumours.^12^, ^13^ Numerous characteristic mutations existed in IVLBCL, such as MYD88 and CD79B, among others.^14^ Given that IVLBCL cells are distributed around blood vessels, a significant amount of ctDNA may be released into peripheral blood after tumour cell death. Prior research has demonstrated the utility of targeted ctDNA sequencing in aiding the diagnosis of IVLBCL.^15^, ^16^ Furthermore, some studies have reported that dynamically monitoring changes in the concentration of ctDNA and the frequency of various mutations could help to assess the efficacy of treatment and enable earlier evaluation of recurrence before clinical symptoms or imaging.^17^, ^18^, ^19^ Therefore, in IVLBCL, the concentration of ctDNA and frequencies of mutations would exhibit synchronous fluctuations as treatment progresses. However, the quality and accuracy differences between ctDNA sequencing and biopsy tissue DNA sequencing remain to be evaluated.

This study aimed to explore the mutation landscape of IVLBCL through targeted plasma ctDNA sequencing and compare the differences in mutation profiles and frequencies between ctDNA and tissue DNA sequencing. Additionally, we conducted dynamic monitoring of ctDNA sequencing results during the treatment process to investigate the potential of ctDNA in evaluating treatment effectiveness. We hope that through this study, we can investigate whether ctDNA can serve as a surrogate for the diagnosis and mutation analysis of IVLBCL, and whether ctDNA is more sensitive and easily accessible than biopsy tissue.

## 2 METHOD

### 2.1 Patients

The local ethical review boards approved this retrospective cohort study, conducted at Peking Union Medical College Hospital. All patients were informed, and the procedure was performed under the Declaration of Helsinki. All patients with IVLBCL were diagnosed by pathological examination via biopsy between February 2015 and October 2023. A total of 34 patients with IVLBCL were included. A total of 37 patients with IVLBCL diagnosed by pathological examination between April 2022 and May 2023 were included to detect the concentration of ctDNA. Patient characteristics, including neurological symptoms, and laboratory data, including haematological, biochemical and immunological findings, were collected from electronic medical records.

### 2.2 Sample collection and DNA extraction

Five millilitres of whole blood was collected by EDTA blood collection tubes then centrifuged within 1 h of collection at 1800×*g* for 10 min at 4≥°C or RT to remove blood cells. The supernatant containing the plasma was removed with special care taken as to not disturb the buffy coat. This was then centrifuged at 16,000×*g* for 10 min to remove any remaining cells. ctDNA was extracted from 2 mL plasma, by digestion in 100 μL proteinase K buffer for 10 min at 37°C followed by purification with the NucleoSpin Plasma XS kit with modified protocols. Tissue genomic DNA of the patients with IVLBCL was purified from the FFPE slides using a QIAamp DNA FFPE Tissue Kit (Qiagen) and from oral swabs using a DNeasy Blood & Tissue Kit (Qiagen). The purified ctDNA and tissue DNA were quantified by a Picogreen fluorescence assay using the provided lambda DNA standards (Invitrogen).

### 2.3 Hybrid selection and ultra‐deep next generation sequencing of ctDNA

The 5′‐biotinylated probe solution was provided as capture probes, the baits target 127 leukaemia and lymphoma‐related genes. One microgram of each DNA‐fragment sequencing library was mixed with 5 μg of human Cot‐1 DNA, 5 μg of salmon sperm DNA and 1 unit adaptor‐specific blocker DNA in hybridization buffer, heated for 10 min at 95°C, and held for 5 min at 65°C in the thermocycler. Within 5 min, the capture probes were added to the mixture, and the solution hybridization was performed for 16–18 h at 65°C. After hybridization was complete, the captured targets were selected by pulling down the biotinylated probe/target hybrids using streptavidin‐coated magnetic beads, and off‐target library was removed by washing with wash buffer. The PCR master mix was added to directly amplify (6–8 cycles) the captured library from the washed beads. After amplification, the samples were purified by AMPure XP beads, quantified by qPCR (Kapa) and sized on bioanalyzer 2100 (Agilent). Libraries were normalized to 2.5 nM and pooled. Deep Sequencing was performed on the HiSeq Sequencing System (Illumina, San Diego, CA) with 2 × 151‐bp paired‐end reads. Cluster generation and sequencing were performed according to manufacturer's protocol.

### 2.4 Sequence alignment and processing

Base calling was performed using bcl2fastq v2.16.0.10 (Illumina, Inc.) to generate sequence reads in FASTQ format (Illumina 1.8+ encoding). Quality control (QC) was applied with Trimmomatic.^20^ High‐quality reads were mapped to the human genome (hg19, GRCh37 Genome Reference Consortium Human Reference 36) using modified BWA aligner 0.7.12^21^ with BWA‐MEM algorithm and default parameters to create SAM files. Picard 1.119 (http://picard.sourceforge.net/) was used to convert SAM files into compressed BAM files which were then sorted according to chromosome coordinates. The Genome Analysis Toolkit^22^ (GATK, version 3.4–0) was modified and used to locally realign the BAMs files at intervals with indel mismatches and recalibrate base quality scores of reads in BAM files.^23^

### 2.5 SNVs/indels/CNVs detections /SV

Single nucleotide variants (SNVs) and short insertions/deletions (indels) were identified using VarScan2 2.3.9^24^ with minimum variant allele frequency threshold set at 0.01 and *p*‐value threshold for calling variants set at 0.05 to generate Variant Call Format (VCF) files. All SNVs/indels were annotated with ANNOVAR, and each SNV/indel was manually checked with the Integrative Genomics Viewer^25^ (IGV). Copy number variations (CNVs) were identified using ADTEx 1.0.4.^26^ Structural variant (SV) were identified by DELLY.^27^ Mutation landscapes of ABC‐type and GCB‐type DLBCL reported in previous study was used to compared in this study.^28^

### 2.6 Statistical analysis

Data were analysed with the Statistical Package for Social Sciences, version 27.0 (SPSS Inc., Chicago, IL, USA). The Mann–Whitney *U*‐test was used to compare median values of data that were not normally distributed. The chi‐square test was used to compare the categorical variable.

## 3 RESULTS

### 3.1 Characteristics of the patients with IVLBCL

A total of 34 patients were enrolled in this study, as presented in Table 1. The median age of the patient cohort was 60.5 years and 17 patients (50%) were male. Among the enrolled patients, 29 patients (85.3%) had an IPI score >3 and 25 patients (73.5%) had a performance status >2. Thirty‐three patients (97.1%) were in stage IV according to the Ann Arbor classification. Notably, B‐symptoms were reported in 29 cases (85.4%). Additionally, bone marrow, CNS and skin involvement were identified in 13, 14 and 13 patients. Serum levels of LDH and IL‐10 were measured, with median concentrations of 1409 (IQR, 417.5–1585) U/L and 322.5 (IQR, 58.6–1000) pg/mL, respectively.

**TABLE 1:** Characteristics of the patients with IVLBCL.

Characteristics	IVLBCL
Total number	34
Age in years, median (range)	60.5 (41–75)
Sex, *N* (%)	
Male	17 (50%)
Female	17 (50%)
Performance status (ECOG)	
0–1	9 (26.5%)
≥2	25 (73.5%)
B symptom	29 (85.4%)
Ann Arbor stage	
IIA	1 (2.9%)
IVA	2 (5.9%)
IVB	31 (91.1%)
IPI score	
Low/intermediate (0–3)	6 (17.6%)
High (4, 5)	29 (85.3%)
Involvement site	
Bone marrow	13 (38.2%)
CNS	14 (41.2%)
Skin	13 (38.2%)
HLH	18 (52.9%)
LDH levels (U/L), median (IQR)	1409 (417.5–1585)
IL‐10 levels (pg/mL), median (IQR)	322.5 (58.6–1000)
CRP levels (mg/L), median (IQR)	70.3 (30.0–124.8)
SF levels (18 μg/L), median (IQR)	922.5 (362.5–2871.0)

Among the 34 patients, four individuals declined chemotherapy for IVLBCL treatment and instead received various doses of steroids along with supportive care. In contrast, the remaining 30 patients received diverse immunochemotherapy regimens, including R‐CHOP with zanubrutinib (*n* = 26), R‐CHOP alone (*n* = 1), R‐CHOP with MTX (*n* = 1), R‐CHOP with zanubrutinib and MTX (*n* = 1), and rituximab with MTX (*n* = 1). Survival analysis was performed on these 30 patients, with a median follow‐up period of 15.2 months (interquartile range, 9.4–23.9 months). Notably, only one patient experienced relapse and subsequently succumbed after 8.0 months. The median progression‐free survival (PFS) for this group was not reached (Figure S1).

### 3.2 Concentration of plasma ctDNA in IVLBCL

**FIGURE 1:**
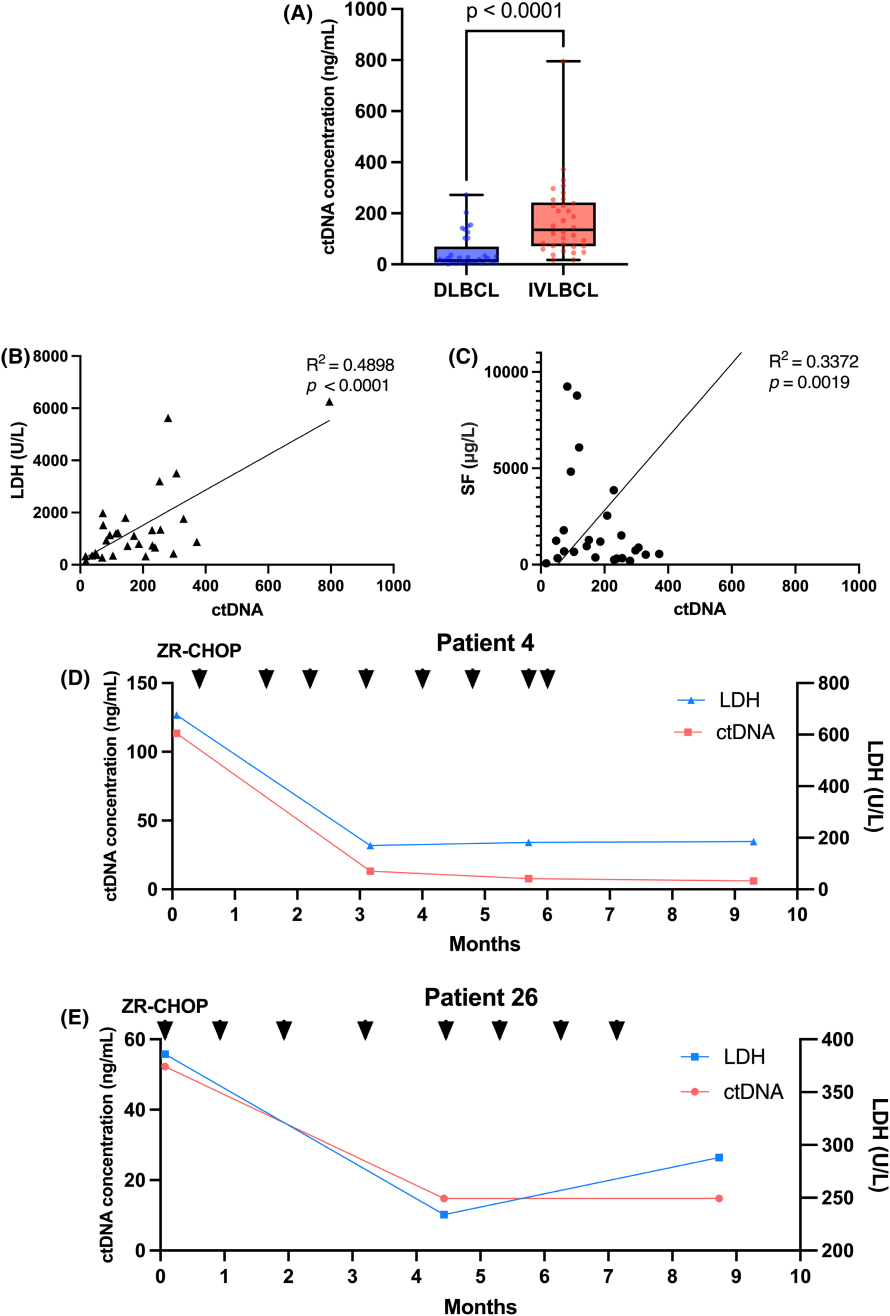
Concentration of plasma ctDNA. (A) The plasma ctDNA concentration of DLBCL and IVLBCL. Compared with DLBCL, IVLBCL had a higher concentration of plasma ctDNA. (B) The concentration of ctDNA was significantly correlated with serum LDH. (C) The concentration of ctDNA was significantly correlated with serum SF. (D, E) Dynamic monitoring of ctDNA concentration and serum LDH level in patients.

To better understand the molecular pathogenesis of IVLBCL, we collected plasma to extract the ctDNA of 34 patients with IVLBCL. The median concentration of plasma ctDNA was 135.0 (IQR, 71.3–242.0) ng/mL (Figure 1A). Plasma ctDNA concentrations of 37 patients with DLBCL were also detected for comparison. The characteristics of patients with DLBCL were shown in Table S1. The concentration of plasma ctDNA extracted from IVLBCL patients was significantly higher than that extracted from DLBCL patients (mean, 172.7 vs. 48.4 ng/mL, *p* < 0.001). The correlation analysis found that the plasma ctDNA concentration was remarkably correlated with LDH and SF levels (Figure 1B,C), but not with CRP levels, IL‐10 levels, bone marrow involvement and complicated HLH (Figure S2). In eight patients undergoing dynamic monitoring with ctDNA concentration, a significant decrease in ctDNA concentration and LDH levels was observed after receiving chemotherapy (Figure 1D,E and Figure S3).

### 3.3 Mutation analysis of ctDNA in IVLBCL

**FIGURE 2:**
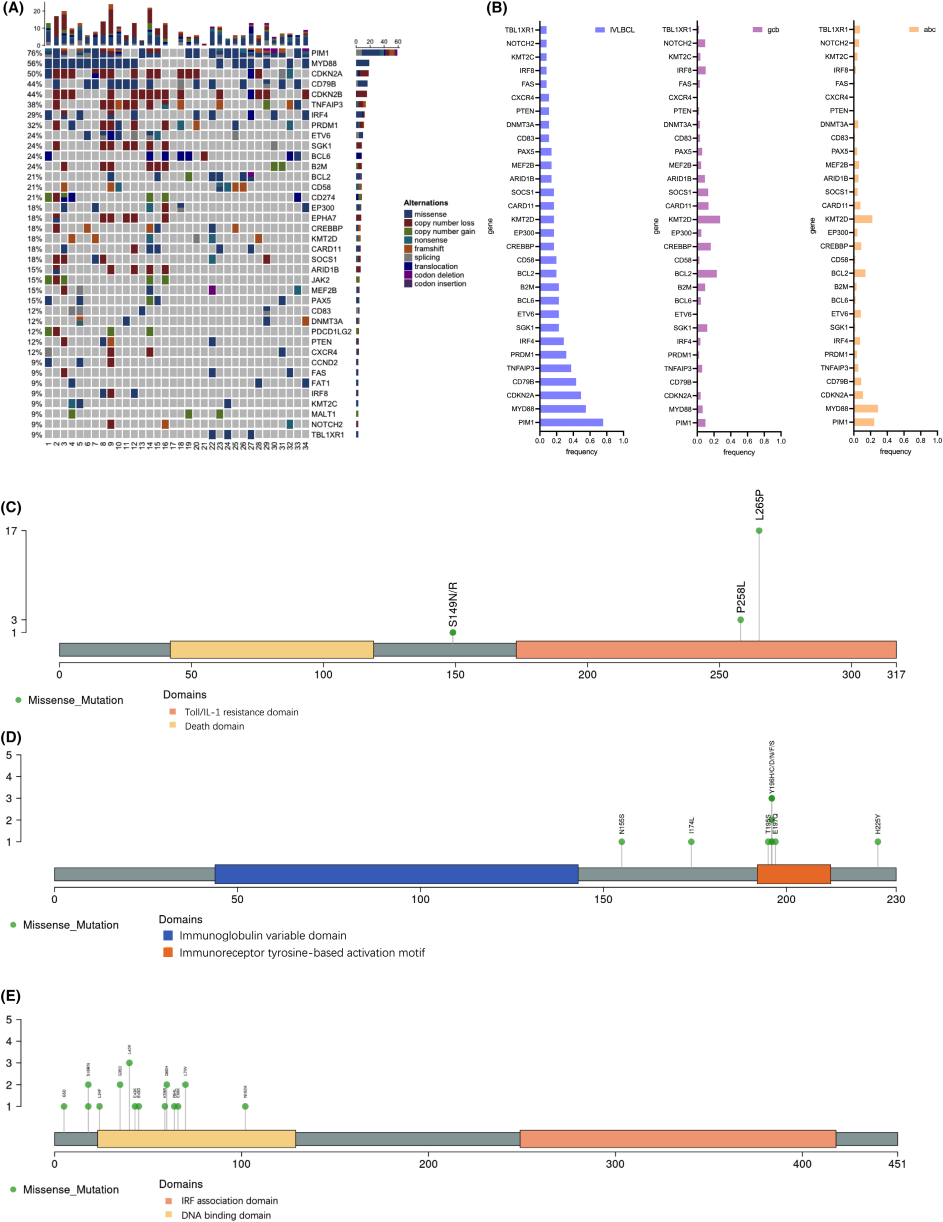
Mutation analysis of ctDNA in IVLBCL. (A) Mutation landscape of ctDNA in IVLBCL. (B) Comparison of mutation landscapes between IVLBCL, ABC‐type DLBCL and GCB‐type DLBCL. (C–E) The locations of the mutations in the MYD88, CD79B and IRF4 gene are shown.

Baseline plasma samples were collected from all 34 patients with IVLBCL and underwent targeted‐panel sequencing including 127 genes (Figure 2A and Table S2). In all 34 patients, mutations were detected in the ctDNA sequencing. Identified mutations were markedly enriched in the B‐cell receptor and NF‐κB signalling pathways, including *MYD88* (56%), *CD79B* (44%), *TNFAIP3* (38%) and *IRF4* (29%). *PIM1* (76%) was the most frequent mutation, which was usually recognized as a somatic hypermutation.^15^ Copy number loss of *CDKN2A* (50%) and CDKN2B (44%) were frequently found, which were related to cell cycle modification. Compared to the mutation landscapes of ABC‐type and GCB‐type DLBCL reported in previous study^28^ (Figure 2B), mutations in IVLBCL such as *PIM1*, *MYD88* and *CD79B* had higher mutation frequencies. Moreover, the mutation landscape in IVLBCL was more similar with that in ABC‐type DLBCL than that in GCB‐type DLBCL.

Since only one patient experienced relapse during the follow‐up, correlation analysis between mutation profile and prognosis could not be conducted in this study. Fisher's precision probability test revealed that compared to patients without CNS involvement, those with CNS involvement had significantly higher mutation rates in *BCL6* (*p* = 0.028) and *CD58* (*p* = 0.011) (Figure S4A,B). Furthermore, compared to patients without haemophagocytic lymphohistiocytosis (HLH), those with HLH had significantly higher mutation rates in *B2M* (*p* = 0.039) (Figure S4C).

In addition, mutations of *MYD88*, *CD79B* and *IRF4*, which were usually considered as driver mutations, were frequently point mutation. Therefore, we further investigated the mutational sites of them. Mutations in *MYD88* were predominantly located in the Toll/IL‐1 resistance domain, with the most frequent mutation being *L265P* (Figure 2C). Recurrent mutations in *CD79B* primarily clustered in the immunoreceptor tyrosine‐based activation motif, and mutations at amino acid positions 195–197 exhibited both high frequency and significant heterogeneity (Figure 2D). Mutations in *IRF4* were dispersed throughout the DNA binding domain, with no specific site demonstrating a markedly higher mutation frequency (Figure 2E).

### 3.4 Mutational comparisons between matched plasma ctDNA and tissue DNA

**FIGURE 3:**
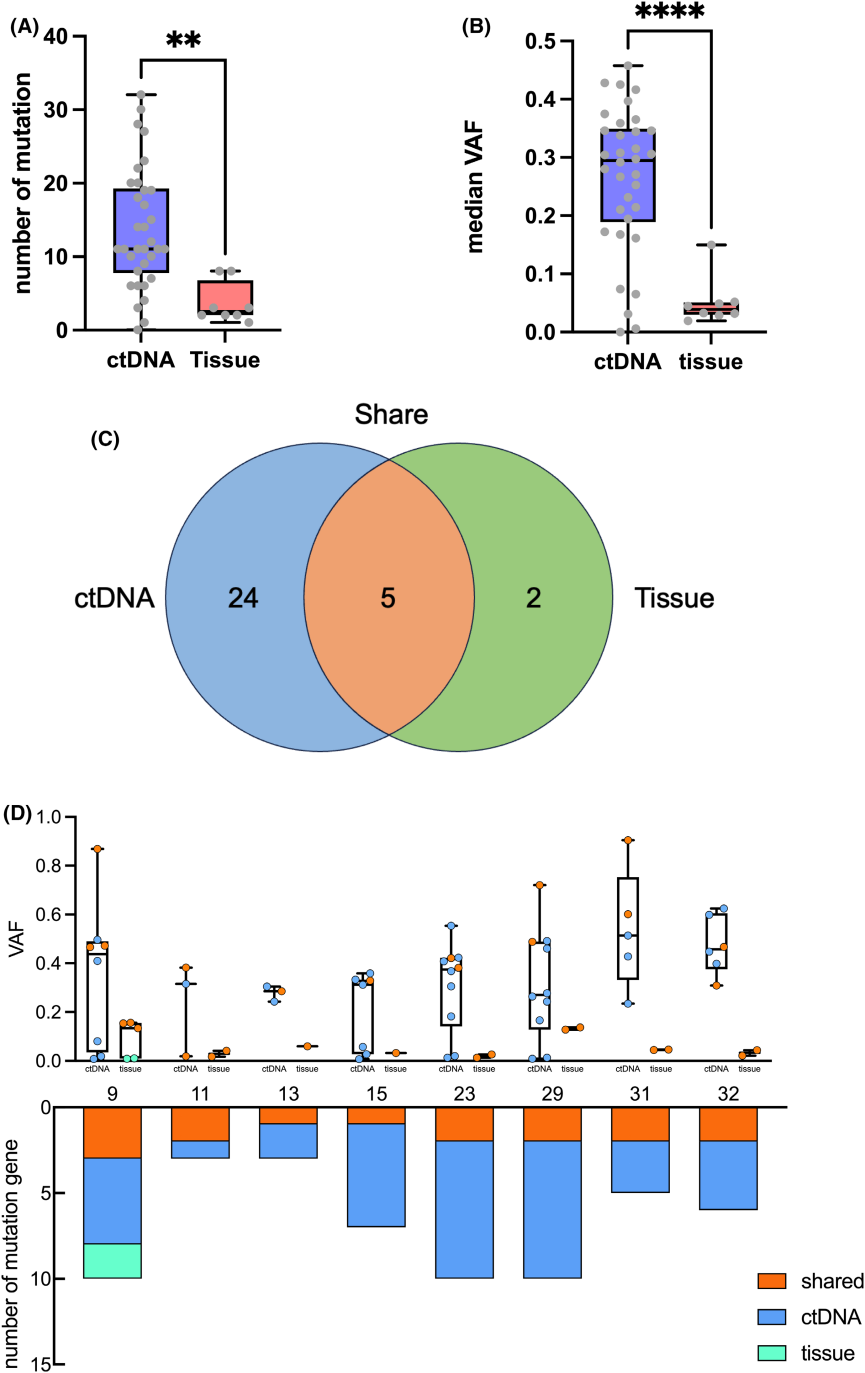
Mutational comparisons between matched plasma ctDNA and tissue DNA. (A) Number of mutations detected in baseline plasma ctDNA and biopsy tissue. Plasma ctDNA had detected significantly more mutations. (B) VAFs of mutations detected in baseline plasma ctDNA and biopsy tissue. VAFs of mutations detected in plasma ctDNA were markedly higher than that detected in biopsy tissue. (C) Number of total mutation genes detected in plasma ctDNA and biopsy tissue. (D) VAFs and number of mutation gene in plasma ctDNA and biopsy tissue of eight patients. ** *p* value < 0.01. **** *p* value < 0.0001.

Paired baseline plasma ctDNA samples and baseline biopsy tissue DNA samples were collected from 8 patients and underwent targeted‐panel sequencing. Among the all 34 patients, biopsy samples from 22 patients were too small for sequencing analysis, while biopsy samples from 4 patients were collected at other institutions and could not be obtained. Therefore, sequencing analysis of biopsy tissue was only conducted for 8 patients. The sequencing result shown that the number of identified mutations in ctDNA were significantly higher than that in tissue DNA (Figure 3A). Moreover, mutations in ctDNA had substantially higher variant allele fraction (VAF) compared with those in tissue DNA (Figure 3B). Most of the mutations detected in tissue DNA were also detected in ctDNA. Additionally, a considerable number of unique mutations were only detected in ctDNA (Figure 3C,D). Thus, these results suggested that plasma can serve as a surrogate for mutational analysis of IVLBCL and is more sensitive and more easily accessible than biopsy tissue.

### 3.5 Dynamic monitor of ctDNA sequencing

**FIGURE 4:**
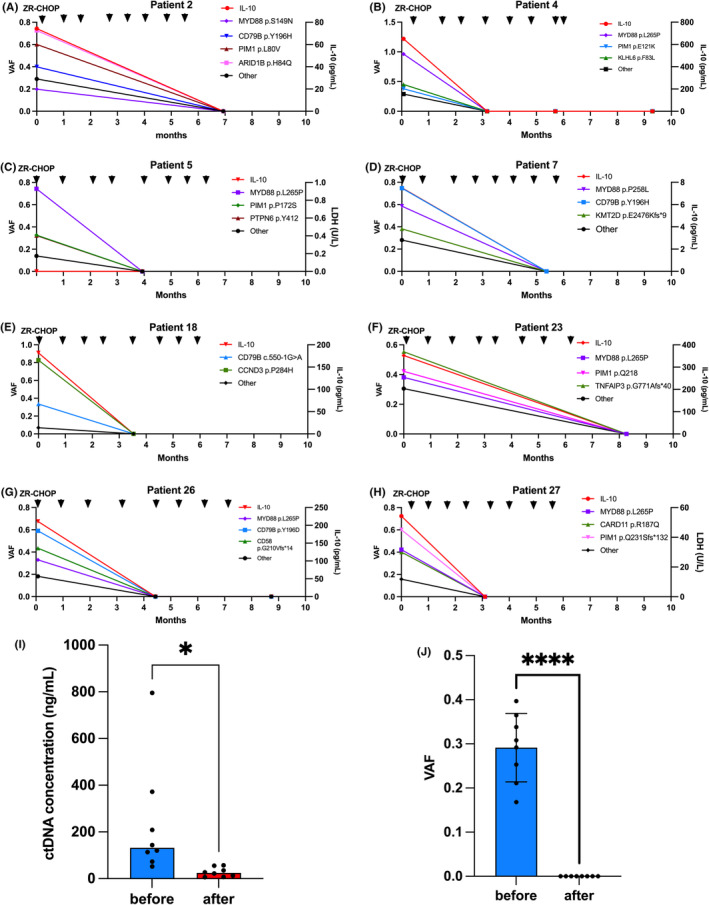
Dynamic change of serum IL‐10 levels and VAFs of mutations in patients. (A–H) Change of VAFs in eight patients. (I) ctDNA concentration of eight patients before and after treatment. (J) Median VAF of eight patients before and after treatment. * *p* value < 0.05. **** *p* value < 0.0001.

In order to dynamically monitor the therapeutic responses, dynamic plasma samplings were performed in eight patients throughout the treatment (Figure 4 and Figure S2). These plasma samples were all sequenced to analyse the change of VAFs of mutations. The ctDNA concentration and VAF decreased significantly after treatment (Figure 4I,J). Moreover, the concentrations of IL‐10 in plasma of these eight patients were also analysed to assist with the evaluation of therapeutic responses. The changes of IL‐10 levels were highly related with the changes of VAFs. These findings indicated that plasma ctDNA sequencing could assist evaluate therapeutic responses in IVLBCL.

## 4 DISCUSSION

To our knowledge, this study represents the first and largest investigation to date, focusing on the mutational profiles of plasma ctDNA and tissue DNA in Chinese patients diagnosed with IVLBCL by employing target‐panel sequencing including 127 lymphoma‐related genes. The results highlight the effectiveness of targeted sequencing of plasma ctDNA for diagnosing IVLBCL and analysing its mutation landscape. Moreover, ctDNA exhibited greater sensitivity in genetic analyses compared to biopsy tissue DNA.

In the analysis of ctDNA concentration, we observed a higher plasma ctDNA concentration in IVLBCL compared to DLBCL. The high plasma ctDNA concentration in IVLBCL may be attributed to the distribution of IVLBCL cells around blood vessels or the multiple involvement of IVLBCL, which often leads to a later tumour staging.^11^ Additionally, ctDNA concentration showed a significant correlation with indicators of disease severity such as LDH and SF. As treatment progressed, a notable decrease in ctDNA concentration was observed. This suggests that ctDNA mainly originated from lymphoma cells surrounding blood vessels and ctDNA may have the ability to serve as an indicator of disease progression and facilitate the monitoring of disease dynamics.

In the genetic analysis of plasma ctDNA, we found frequent mutations in genes related to the BCR/NF‐KB signalling pathway in IVLBCL. Furthermore, compared to the ABC and GCB subtypes of DLBCL, IVLBCL had higher mutation frequencies in *MYD88* (56%), *CD79B* (44%), *TNFAIP3* (38%) and *IRF4* (29%).^28^ Shimada K et al. has reported the mutational signatures of IVLBCL using whole‐exon ctDNA sequencing in a Japan cohort (*n* = 21), finding that the mutation frequencies of genes related to the BCR/NF‐KB signalling pathway such as *MYD88*, *CD79B*, *TNFAIP3* and *IRF4* were 57%, 67%, 24% and 38%, respectively. And PIM1 mutation was detected in 95% patients. Gonzalez‐Farre et al. investigated 15 Caucasian patients with IVLBCL, finding that mutation frequencies of *MYD88*, *CD79B* and *IRF4* were 53%, 53% and 27%, respectively.^14^ The results of Japan cohort and Caucasian cohort were comparable to the finding observed in our study. Other Western study also reported similar frequencies of *MYD88* and *CD79B* mutations as that in our study.^10^ Based on these results, the mutation profiles among IVLBCL patients from different populations appear to be relatively similar, without significant differences between races.

Correlation analysis indicated that among patients with CNS involvement, there was a significantly higher frequency of *BCL6* mutation and lower frequency of *CD58* mutation. Among patients with concurrent HLH, there was a significantly higher frequency of mutations in β2‐microglobulin (*B2M*). In our study, we observed that the majority of *BCL6* mutations were translocations, which can upregulate its expression and indicating a poor prognosis.^29^ Moreover, most *CD58* mutations identified were nonsense and frameshift mutations. Expression of CD58 and ligation to CD2 is required for anti‐tumour immunity. Defects of *CD58* promote immune evasion by diminishing T‐cell activation, impairing intratumoral T‐cell infiltration and proliferation, and concurrently increasing PD‐L1 protein stabilization.^30^ These mutated genes potentially contribute to the mechanism of central nervous system involvement in lymphoma. Nevertheless, further research is needed to explore the potential role of *B2M* in the pathogenesis of HLH. Consistent with previous studies,^16^ in IVLBCL, mutations in *CD79B* are primarily happened near the 196th amino acid residue in the ITAM domain, while mutations in *MYD88* were mainly the L265P mutation existing in the TIR domain. Additionally, in this study, we also found that mutations in *IRF4* are predominantly clustered in the DNA‐binding domain.

In this study, we compared the mutation analysis results of plasma ctDNA and tissue DNA from eight patients. The other patients had insufficient pathological tissue for extracting an adequate amount of DNA for sequencing analysis. We found that ctDNA detected more mutations than tissue DNA, and the detected mutations had higher VAFs. In our study, the majority of mutations were detected by plasma ctDNA sequencing. We hypothesize that the limited presence of tumour cells in IVLBCL resulted in a lower concentration of tumour DNA extracted from biopsy samples, making it challenging to detect the majority of mutations. A significant portion of mutations detected in ctDNA were not found in tissue DNA. This indicates that DNA produced by lymphoma cells surrounding blood vessels is more enriched in plasma. Moreover, using plasma ctDNA for mutation analysis provides a more comprehensive analysis of the mutation profile of IVLBCL compared to tissue DNA. Considering that IVLBCL typically requires multiple repeated biopsies to detect tumour cells and confirm diagnosis, collecting plasma ctDNA is easier than collecting tissue DNA. Therefore, ctDNA can serve as a surrogate for the diagnosis of IVLBCL and mutation analysis of IVLBCL and ctDNA is more sensitive and easily accessible than biopsy tissue.

During the treatment process, we also collected and sequenced plasma ctDNA repeatedly from eight patients. However, due to the short follow‐up period in this study, changes in ctDNA in relapsed patients were not observed. Therefore, larger‐scale and longer‐term studies are needed to validate the sensitivity, specificity and efficacy of ctDNA monitoring for assessing treatment efficacy, detecting small lesions and predicting relapsed. Both the concentration of ctDNA and the changes in VAF of mutated genes were correlated with treatment response. The ctDNA concentration and VAF decreased significantly after treatment. Due to the significant decrease in ctDNA concentration and mutation VAF after treatment, if we shorten the detection intervals post‐treatment to monitor the time when ctDNA concentration and VAF begin to decline, this might serve as a potential early biomarker for detecting the onset of treatment efficacy. Due to the relatively short follow‐up period in this study, we are currently unable to address whether the mutation profile is associated with prognosis.

In the survival analysis, only one patient experiencing relapse was observed. As a rare tumour, IVLBCL have a relatively good prognosis. In another recent study using the R‐CHOP combined with high‐dose methotrexate plus intrathecal chemotherapy regimen, the 1‐year survival rate was 94.6%,^31^ which was very close to the 95.7% 1‐year survival rate observed in our study. In another study of us on IVLBCL, we used the R‐CHOP combined with MTX regimen and also achieved similarly good therapeutic outcomes.^32^ Moreover, among the 30 patients in our study, 26 received R‐CHOP plus zanubrutinib. Zanubrutinib is a BTK inhibitor reported to be effective for B‐cell lymphomas with MYD88 and CD79b mutations, double expression of MYC and BCL2/BCL6, and CD5 expression.^33^, ^34^, ^35^ These mutations and expression are highly prevalent in IVLBCL. Therefore, Zanubrutinib may have contributed to these positive outcomes. Due to the relatively short follow‐up period in our study, with only seven patients followed for more than 2 years, the limited follow‐up time may also explain why only one patient experiencing relapse was observed.

This study has some limitations. First, due to the rarity of IVLBCL, our study included a total of only 34 patients. Other studies analysing the mutational landscape of IVLBCL also included a relatively limited number of patients (*n* = 21, *n* = 15 and *n* = 9).^14^, ^15^, ^16^ The number of biopsy pathological tissue samples was also insufficient. Therefore, the conclusions of this study need further validation in larger‐scale studies. Second, the median follow‐up time of this study was 15.2 months, which was relatively short. In survival analysis, the median PFS and OS were not reached. Additionally, in the dynamic monitoring of ctDNA, changes in ctDNA were not observed in relapse patients. We cannot provide the mutation or dynamics factors associated with relapse risk. Therefore, longer follow‐up is needed to obtain data related to relapse, address relapse‐related issues and analyse the survival outcomes of IVLBCL, treatment efficacy, and the effectiveness of dynamic ctDNA monitoring. Currently, we are conducting a study using ZR‐CHOP regimen to treat IVLBCL and have achieved some early results.^36^ Efficacy and survival‐related questions may be addressed by the future findings of this study (NCT04899570).

In this study, we revealed that plasma ctDNA can assist in diagnosing IVLBCL and analysing the mutational landscape of IVLBCL. Targeted sequencing of plasma ctDNA was more sensitive and easily accessible than biopsy tissue. Dynamic monitoring of ctDNA can help assess treatment efficacy.

## AUTHOR CONTRIBUTIONS

**Chao Chen:** Conceptualization (equal); data curation (equal); investigation (equal); methodology (equal); visualization (equal); writing – original draft (equal). **Yiao Di:** Data curation (equal); formal analysis (equal); investigation (equal); methodology (equal). **Zhe Zhuang:** Software (equal); supervision (equal); validation (equal). **Hao Cai:** Data curation (equal); formal analysis (equal). **Congwei Jia:** Methodology (equal); resources (equal); software (equal). **Wei Wang:** Data curation (equal); formal analysis (equal); investigation (equal). **Danqing Zhao:** Investigation (equal); methodology (equal); project administration (equal); resources (equal). **Wei Chong:** Resources (equal); software (equal); supervision (equal); validation (equal). **Wei Zhang:** Formal analysis (equal); methodology (equal); project administration (equal); writing – review and editing (equal). **Daobin Zhou:** Investigation (equal); project administration (equal); validation (equal); writing – review and editing (equal). **Yan Zhang:** Funding acquisition (equal); project administration (equal); writing – review and editing (equal).

## FUNDING INFORMATION

This study was funded by the National High Level Hospital Clinical Research Funding [2022‐PUMCH‐A‐192, 2022‐PUMCH‐B‐029] and Capital's Funds for Health Improvement and Research [2024‐2‐4011].

## CONFLICT OF INTEREST STATEMENT

The authors have no competing financial interests or other conflicts of interest to disclose.

## CONSENT

All patients provided written informed consent prior to enrolment.

## PERMISSION TO REPRODUCE MATERIAL FROM OTHER SOURCES

The article didn't reproduce material from other sources.

## Supporting information


**Data S1:** Supporting Information.

## Data Availability

The data that support the findings of this study are available from the corresponding author upon reasonable request.
