# Supplementary material for: Plasma circulating tumour DNA is a better source for diagnosis and mutational analysis of IVLBCL than tissue DNA
Source: J Cell Mol Med. 2024 Jul 25;28(14):e18576. doi: 10.1111/jcmm.18576 (PMC11272604; doi:10.1111/jcmm.18576)
Supplement: Supplementary file 1 — Data S1: Supporting Information. [file JCMM-28-e18576-s001.docx]

| Characteristics | DLBCL |
| --- | --- |
| Total number | 37 |
| Age in years, median (range) | 60.0 (41-66.5) |
| Sex, N (%) |  |
| Male | 22 (59.5%) |
| Female | 15 (40.5%) |
| Ann Arbor stage |  |
| I | 4 (10.8%) |
| II | 4 (10.8%) |
| III | 7 (18.9%) |
| IV | 22 (59.5%) |
| IPI score |  |
| Low/intermediate (0–3) | 29 (17.6%) |
| High (4, 5) | 8 (85.3%) |
| Number of patients with detected mutations | 30 (81.8%) |

Table S1. Characteristics of the patients with DLBCL.

| ALK | BIRC3 | CD79B | FAS | KDR | MYC | PIM1 | STAT3 | XPO1 |
| --- | --- | --- | --- | --- | --- | --- | --- | --- |
| AKT1 | BTK | CD83 | FAT1 | KIT | MYD88 | PLCG2 | STAT6 | IGH |
| AKT2 | BRAF | CD58 | FBXO11 | KLHL6 | NF1 | POT1 | SYK | IGK |
| APC | CALR | CIITA | FBXW7 | KMT2A | NFKBIA | PRDM1 | TBL1XR1 | IGL |
| ARID1A | CARD11 | CREBBP | FOXO1 | KMT2C | NFKB1 | PTEN | TCF3 | TRB |
| ARID1B | CCND1 | CTLA4 | GNA13 | KMT2D | NFKB2 | PTPN6 | TET2 | TRA |
| ARID2 | CCND2 | CTNNB1 | HRAS | KRAS | NFKBIE | PTPRD | TNFAIP3 | TRG |
| ATM | CCND3 | CXCR4 | ID3 | LYN | NOTCH1 | RB1 | TNFRSF14 |  |
| ATR | CDKN1B | CYLD | IKZF1 | MALT1 | NOTCH2 | ROS1 | TP53 |  |
| B2M | CDKN2A | DDX3X | INPP5D | MAP2K1 | NRAS | SETD2 | TP63 |  |
| BCL10 | CDKN2B | DNMT3A | IRF4 | MAP3K14 | PAX5 | SGK1 | TP73 |  |
| BCL2 | CDKN2C | DTX1 | JAK1 | MDM2 | PDCD1LG2 | SF3B1 | TRAF2 |  |
| BCL6 | CD274 | EP300 | JAK2 | MED12 | PIK3R1 | SOCS1 | TRAF3 |  |
| BCOR | CD70 | ERBB4 | JAK3 | MEF2B | PIK3CA | SPEN | TRAF5 |  |
| BCORL1 | CD79A | EZH2 | KDM6A | MTOR | PIK3CD | SMARCA4 | NSD2 |  |

Table S2. 127 leukemia and lymphoma-related targeted genes.


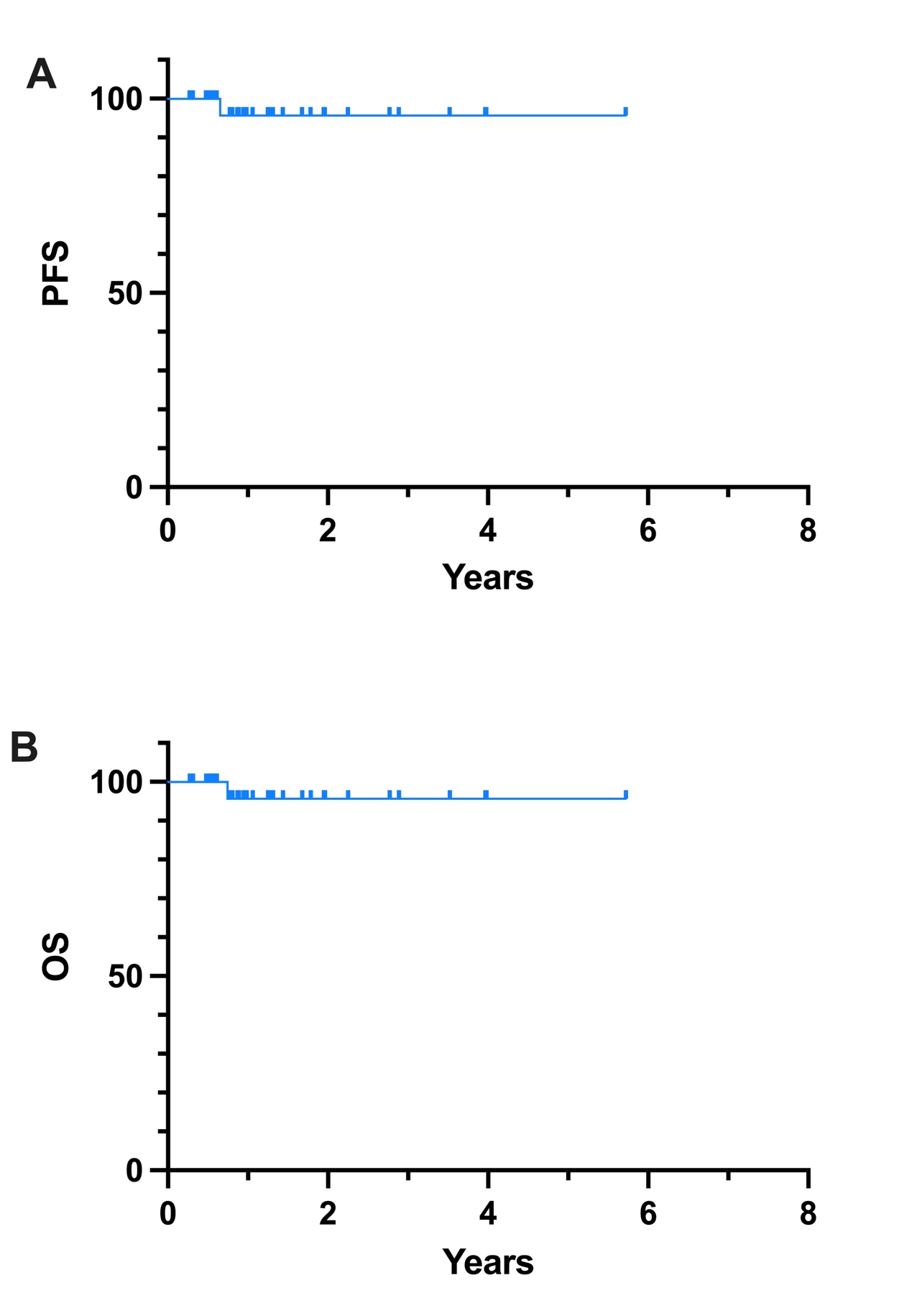


Figure S1. Survival analysis of 30 patients receiving immunochemotherapy. (A) PFS of patients receiving immunochemotherapy. (B) OS of patients receiving immunochemotherapy.


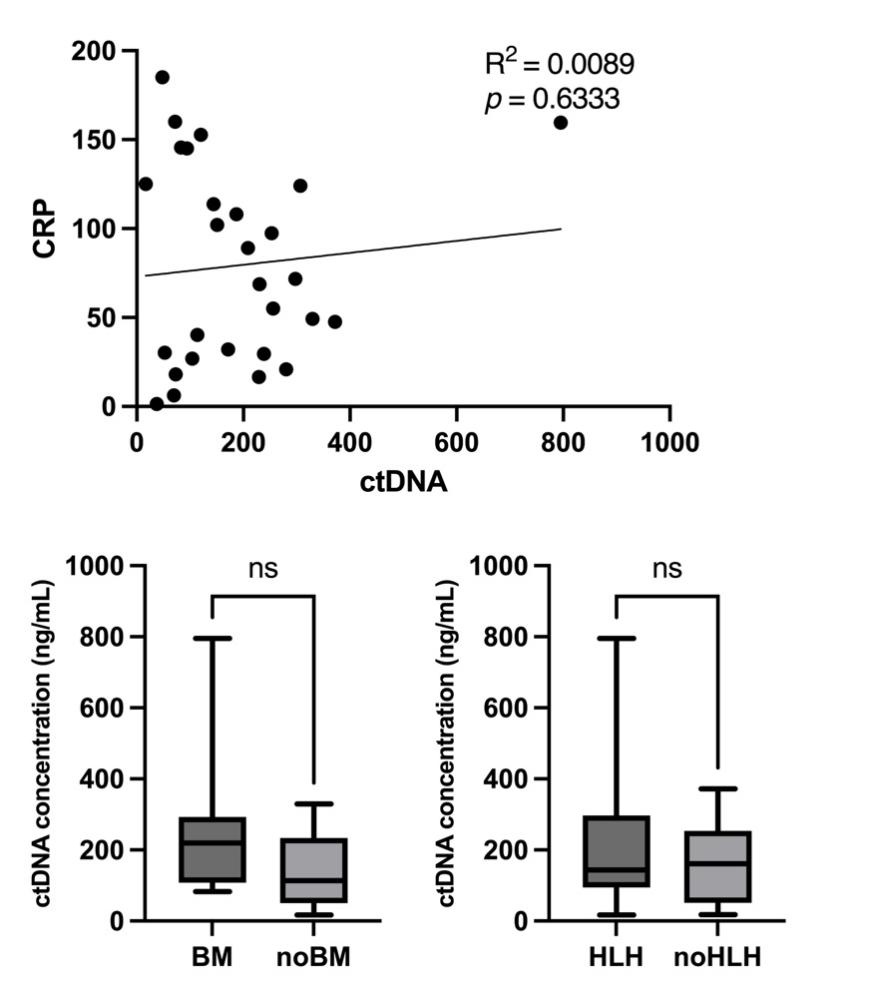


Figure S2. Concentration of plasma ctDNA (A) The concentration of ctDNA was not significantly correlated with serum LDH. (B) The concentration of ctDNA was not significantly different in patients with bone marrow involvement and patients without bone marrow involvement. (C) The concentration of ctDNA was not significantly different in patients with HLH and patients without HLH.


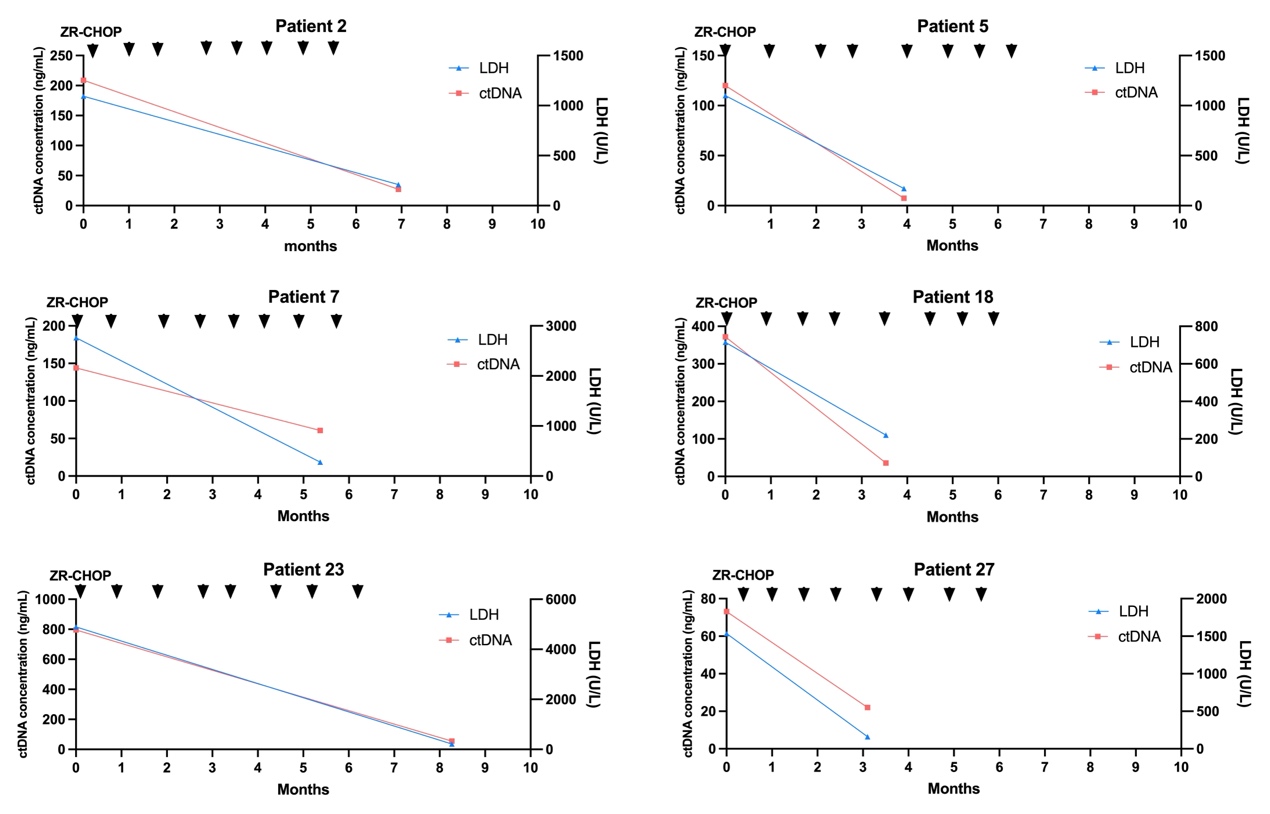


Figure S3. Dynamic monitoring of ctDNA concentration and serum LDH level in patients.


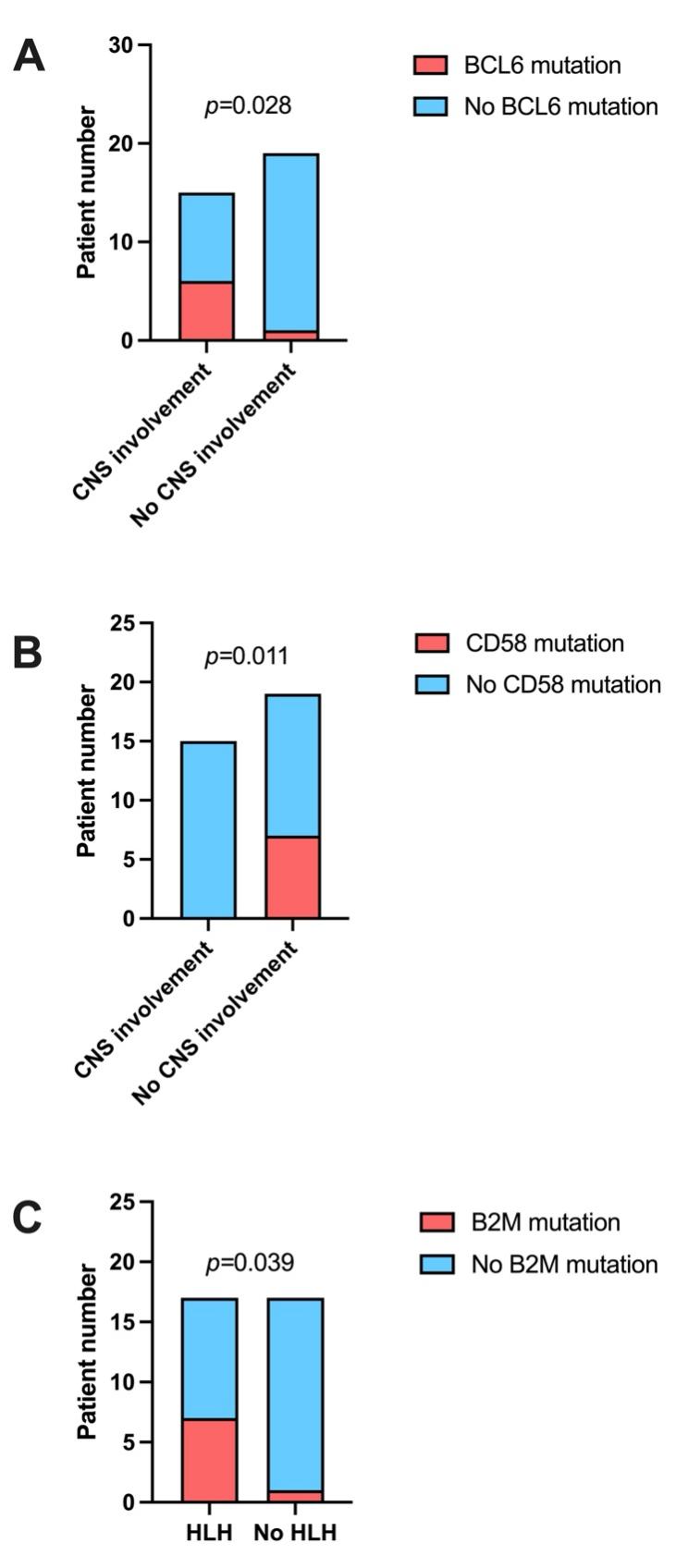


Figure S4. (A) BCL6 mutation in patient with or without CNS involvement. (B) CD58 mutation in patient with or without CNS involvement. (C) B2M mutation in patient with or without CNS involvement.
